# Kids Save Lives – The kids’ and teachers’ view: How school children and schoolteachers would alter a BLS course designed by specialists

**DOI:** 10.1016/j.resplu.2024.100731

**Published:** 2024-08-01

**Authors:** C. Andreotti, M. Kolbe, V. Capon-Sieber, D.R. Spahn, J. Breckwoldt

**Affiliations:** a University Hospital Zurich, Institute of Anesthesiology, Switzerland; b Simulation Center, University Hospital Zurich, Switzerland; c Institute of Education, Dept. for Research on Learning, Instruction, and Didactics, University of Zurich, Switzerland

**Keywords:** Basic life support, Basic life support training, Schoolchildren, Schoolteachers, Kids-Save-Lives, Video-stimulated recall, Delta-Plus method

## Abstract

**Background:**

Training schoolchildren in basic life support (‘Kids-Save-Lives’ training) is widely believed to improve outcomes from out-of-hospital cardiac arrest. Numerous programmes have been launched, but to our knowledge, neither children nor schoolteachers have been directly involved in designing these courses. This is unfortunate, as it is well-known that children (as the target goup of training) learn differently from adults. We therefore sought to explore the view of schoolchildren and their teachers on the design of a ‘Kids-Save-Lives’ course.

**Methods:**

We designed a state-of-the-art, 90-min BLS training and delivered it to all 13 classes of a secondary community school (children aged 12–16). Directly after each training, we performed Video-Stimulated Recall (VSR) with 2 children and 2 schoolteachers. For VSR, we presented video sequences from defined sections of the training and related semi-structured questions to these sections. The interviews were audio-recorded, transcribed, and analysed using qualitative content analysis.

**Results:**

Twenty-four children and 24 teachers participated in the VSR. The overall satisfaction with the training was very high. Participants especially appreciated the brief theoretical introduction using a video, the high practical involvement, and the final scenario. Children suggested the program could be improved by better linking the video to the children’s world, increasing excitement and action, and limiting the group size in the final scenario. Teachers suggested incorporating more theoretical background, using terms and language more consistently, and better integrating the program into the school curriculum.

**Conclusions:**

Although very satisfied with a state-of-the-art ‘Kids-Save-Lives’ training, children and teachers made important suggestions for improvement.

## Background

Bystander cardiopulmonary resuscitation (B-CPR) substantially improves survival after out-of-hospital cardiac arrest.^1^, ^2^, ^3^ Therefore, resuscitation training of the broad population, particularly of schoolchildren, is regarded as a key strategy for saving more lives.^4^, ^5^ ‘Kids-Save-Lives’ programmes have been widely implemented and have been shown to improve CPR knowledge,^6^ skills 7, 8, 9 and self-confidence.^10^ Children trained in schools have in turn passed on their CPR skills to their parents and other relatives.^11^, ^12^

A great variety of ‘Kids-Save-Lives’-programmes have been created.^8^, ^13^, ^14^, ^15^, ^16^, ^17^ However, the vast majority of courses were designed by specialists in traditional resuscitation training, and only very few courses specifically referred to the pedagogical background of children’s education.^16^ Research shows, however, that learning programs designed for adults cannot be directly translated to children and adolescents. For example, adults tend to be more internally motivated and problem-oriented in their learning.^18^, ^19^ In contrast, children and adolescents learn with more playful or competitive approaches and have been shown to be more ‘sensation seeking’.^20^, ^21^ Future course designs could thus be improved by examining the authentic experiences of the target group of learners. Furthermore, involving schoolteachers, as experts in teaching a specific age-group, will provide important insight into opportunities for improvement as well as starting points for collaborative working.

To approach the questions above, we delivered a state-of-the-art ‘Kids-Save-Lives’ training in a secondary school (the most popular age group for these programs) and collected structured feedback from schoolchildren and their teachers. To gather more precise information, the research relied on the methodology of Video-Stimulated Recall (VSR). In VSR, short video sequences taken from specific parts of an event are presented to the interviewees to enhance and focus the recall and to stimulate deeper (and more emotional) reflections on the topic.^22^, ^23^, ^24^ We combined VSR with the ‘Plus-Delta’ debriefing approach,^25^ which utilizes the participants’ first rating of a situation as a starting point to let them further develop ideas to improve a subject or performance. This process encourages interviewees to develop creative solutions rather than remaining in a state of judgement, and the approach is especially effective when interviewing individuals who were exposed to a new subject.^25^

Our objective was to explore how training could be improved from the perspective of the children and their teachers, thus allowing courses to be better tailored to their specific needs. Barriers to implementing the trainings could be reduced, including the perceived lack of competence in CPR-training, which is often cited as a barrier by schoolteachers.^26^

## Methods

### Sample and ethics

From January to September 2020, we delivered the ‘Kids-Save-Lives’-training to all 13 classes of a secondary community school in the Canton of Zurich, Switzerland. The children’s ages ranged from 12 to 16 years and the average class size was 21 children. Educator-participant ratios ranged from 1:5 to 1:8. We conducted the training in the Simulation Center of the University Hospital Zurich. The research followed the Sim-CONSORT framework.^27^

All participants, the teachers and the parents of the children, gave written permission for the study. Information that could have identified specific persons or institutions was anonymized during the transcription process. The study was granted exemption by the Ethical Committee of the Canton of Zurich, BASEC Req-2019-01243.

### Design of the training

We developed the ‘state-of-the-art’ training based on the literature,^14^, ^28^, ^29^ and current ERC guidelines.^30^ Key learning objectives were defined as: to recognize cardiac arrest, to perform sufficient chest compressions, to use an automated external defibrillator (AED), and to minimize the fear of doing harm. The 90-minute training placed a distinct focus on skills and encompassed ‘introduction & theory’, ‘training chest compressions & AED’, and ‘scenario practice’.

### Delivery

The training was delivered by experienced instructor-educators certified by the Swiss Resuscitation Council without a specific background in training school children. In the introduction section for the whole school class, the expectations and previous experiences of the children were briefly explored before theoretical information was provided in a 6-min video showing an adult cardiac arrest (CA) scenario played by actors. The video highlighted recognizing CA, shouting for help, conducting the alarm call, providing chest compressions and applying an AED.

The second part of the course lasted 30 min and focussed on applying that knowledge. We divided the classes into groups of six with one designated educator. Within these groups, the children worked in pairs on one manikin (Brayden, https://www.aerohealthcare.co.uk), taking turns during chest compressions. The manikins had a built-in feedback system displaying red lights in the carotid areas and the forehead to indicate adequate (brain) perfusion if chest compressions were sufficient. We used a metronome to guide children towards the appropriate compression rate. Subsequently, the educators switched to the song ‘*Stayin’ Alive*’ by the band ‘Bee Gees’ to keep up motivation. Then, an AED (ZOLL AED 3 Trainer, Zoll Medical, Switzerland) was introduced. In groups of three to four, the children practiced operating the device and integrating the procedure into ongoing chest compressions. The educators provided feedback until performance was sufficient. To reduce complexity, we intentionally did not address ventilations. Throughout the process, we emphasized the key message: ‘*The only thing you can do wrong is doing nothing*’.

In the third part of the training, again lasting 30 min, we simulated two real-life scenarios in which a real person simulating unconsciousness was found e.g., in the restrooms. The scenario then immediately switched to a manikin in CA (AmbuMan Instrument, AMBU, Dietikon, Switzerland) which was rescued by a team of children. The class was divided into groups of 8–12 pupils with the same two scenarios each. One half of the group was active the other half acted as observers. We deliberately did not assign team leaders. At the end of a scenario, the groups received a brief feedback on their performance.

### Video-stimulated recall (VSR)

Immediately after finishing the training, two children and two schoolteachers, as a group, participated in a 30 min Video-Stimulated Recall (VSR) session,^22^, ^23^, ^24^ working with interviewers who had not been instructing during the training. We collected VSR for four aspects of the course: overall impression, introduction, skills training and the final scenario. At the beginning of each VSR section we presented a 60-second video sequence which had been recorded during the starting phase of that specific course part. Then, each interviewee rated his or her satisfaction with that part of the course using a verbal Likert-like scale from 1 (worst) to 10 (best). Thereafter, interviewees were asked for suggestions on how to improve this part of the course (‘Plus-Delta’ approach**^25^**). To reduce bias through adults’ views, the children were interviewed before the teachers. The interviews were audiorecorded, transcribed verbatim using the software ‘F5′, version 7.0.1. (Audiotranskription, Marburg, Germany) and analyzed using qualitative content analysis.^31^ All information that could have identified specific persons or institutions was anonymized.

### Coding of comments

Three researchers with expertise in CPR training who were not involved in the training sessions independently analyzed three transcripts (20% of the raw material) to derive the main categories. Final consent was reached by discussion. Subsequently, the first author (CA) identified all comments relevant to the research question, coded them according to the main categories and then formed and refined appropriate sub-categories.

### Sample size and statistics

As common in qualitative research, sample size estimation was based on the criterion of ‘saturation of information’, or 'information power',^32^ in which a sample size is considered sufficient once a new interview generates less than 10% new information compared to the previous interviews. We assumed adequate information power was reached at three classes per school year and set the minimum sample size at nine VSR sessions. Given the qualitative nature of the study, we did not calculate quantitative statistics. For the descriptive parameter of satisfaction (used for the Delta-plus method), we provide median, minimum (min) and maximum (max) values.

## Results

Twenty-four schoolchildren and 24 schoolteachers from 12 of the 13 courses participated in the VSR; the average session duration was 25–30 min. Saturation of information was reached at 8–10 VSR sessions. The following main categories were derived by the three independent researchers: (i) course structure, (ii) introductory video, (iii) skills training and (iv) scenario. We present the interviewees’ comments and suggestions for improvement according to these main categories.

### Course structure

Overall, the interviewees rated the course structure with a median of 9 points (min 7, max 10). Eleven interviewees stated that they found the course format appropriate, including overall length, introduction with video, and the distribution between theory and practice.

For improvement, seven children would have preferred having more time for practice, in part because they enjoyed practicing together with their classmates and also to ‘*better retain skills*’. Children also requested that the course should be repeated to enhance retention. Some interviewees (n = 9) proposed smaller group sizes for practicing.

As a further area for improvement, wording and language were mentioned. Four children and six teachers suggested using more consistent, uniform wording and replacing medical terms and clinical jargon with everyday language. Seven teachers suggested providing a glossary of the most important medical terms before the course to prepare the children on a knowledge base (see also, suppl. material 1). Teachers also asked for a uniform use of idiom, preferably High German instead of dialect, as children with a migration background with limited comprehension skills would not necessarily make their difficulties apparent in the group.

Another point for improvement was to discuss more basic theoretical content at the beginning of the course (suggested by three children and nine teachers). This related mainly to the purpose of resuscitation and the mechanisms of cardiac arrest.

At the beginning of the course, some children reported experiences related to cardiac arrest in their families or in the public sphere. The statements revealed an evident fear and apprehension towards such serious topics, due to which schoolteachers suggested establishing the learning objectives more explicitly at the beginning of the course (such as highlighting the message of *‘The only thing you can do wrong is doing nothing‘*).

### Introductory video

The interviewees rated the overall usefulness of the educational video with a median of 9 (min 7, max 10). The majority (n = 34) found the video informative and exciting. Teachers mentioned that the video ‘*caught the children’s attention by creating an exciting introduction*’ and ‘*activated an additional, visual channel’ for ‘a first insight into the upcoming training*’.

The area for improvement mentioned most by children and teachers (n = 25) was the lack of realism (e.g., *the AED was available on-site*, or the *rescuers arrived too quickly*). Interviewees found the video should be made more exciting and realistic by e.g., moving the scene to the outside (to a well-known spot of the city, or ‘*a cool location*’), also in order to produce more tension and thrill (‘*a shock effect*’). Several children stated it would increase their motivation if children and adolescents were protagonists. Children wanted the video to show how the AED was deployed and how the local police and emergency medical services were involved. As a further improvement, teachers found that a narrator in the video could convey theoretical knowledge and a ‘remember’ field at the end could summarize the most important information.

### Skills training

The training of BLS/AED skills was rated with a median of 9 (min 7, max 10). One of the most valued points was the close support by the educators. The children found the continuous feedback particularly helpful as they were ‘*corrected immediately in case of incorrect actions*’. In addition, they highly valued the built-in feedback of the manikins with lights indicating adequate cerebral blood flow. Children and teachers both found it helpful to use a metronome which was later substituted by the song beat. Children stated that the tools ‘*helped them keep the rhythm*’ while ‘*the music was also very motivating*’.

Regarding manikins, four children suggested designing them to be more realistic (such as obese, female, or children), or to address whether and how a female victim should be freed from clothes at her upper body. Two children explicitly stated that typically ‘*the obese people were more likely to suffer cardiocirculatory arrests and were thus more likely to be our patients*’. For the skills training, some children wished to introduce competition among each other to achieve the best possible performance. Finally, schoolteachers stated they would have liked to help during skills teaching, and that they felt capable of delivering the course by themselves.

### Training scenario

The scenario was also rated with a median of 9 points (min 8, max 10). Generally, the scenario was seen as the best way to practice realistic situations (n = 32) by directly incorporating all relevant steps (recognition of arrest, alarm call, chest compressions, AED use). Five children explicitly stated that the ‘*scenario had given more security*’ and ‘*made the subject less scary*. Three children found the scenario had promoted teamwork by allocating the different tasks. Some teachers mentioned that the learning progress of the children became apparent during the scenario. However, the scenario also gave rise to more complex questions, mainly related to situational circumstances. For example, children asked how to move a victim who fell on the stairs without risking back injury, or how an AED should be handled under wet conditions.

Improvements were suggested, including making the scenario more serious (*n* = 12) and thus inducing more emotional involvement for the participants. Interviewees suggested building in moments of surprise (e.g., a person suddenly appearing and causing disturbance), playing scenarios in different locations (e.g., outdoors), or involving bystanders. For the format, nine children recommended reducing the group sizes. They argued that ‘*some children just stood there*’ and only a few had role assignments and could perform tasks. Finally, children and teachers preferred debriefings that were more extensive.

## Discussion

In this study, we showed that a state-of-the-art CPR training for school children was well received by the children and their teachers. This replicates previous findings.^7^, ^14^, ^33^ However, using VSR and the ‘plus-delta’ approach enabled deeper insights into how recipients would alter content and formats. Overall, children would enhance the realism and excitement of all course elements. In addition, teachers, and children, would improve the integration of theory and practice by teaching more background knowledge beforehand at school. Further, teachers and children would prefer using consistent language and technical terms. As a final important point, teachers expressed their confidence in their ability to deliver Kids-Save-Livves courses themselves. In the following sections, we discuss these main findings and their implications.

### Realism of content and excitement

**Fig. 1:**
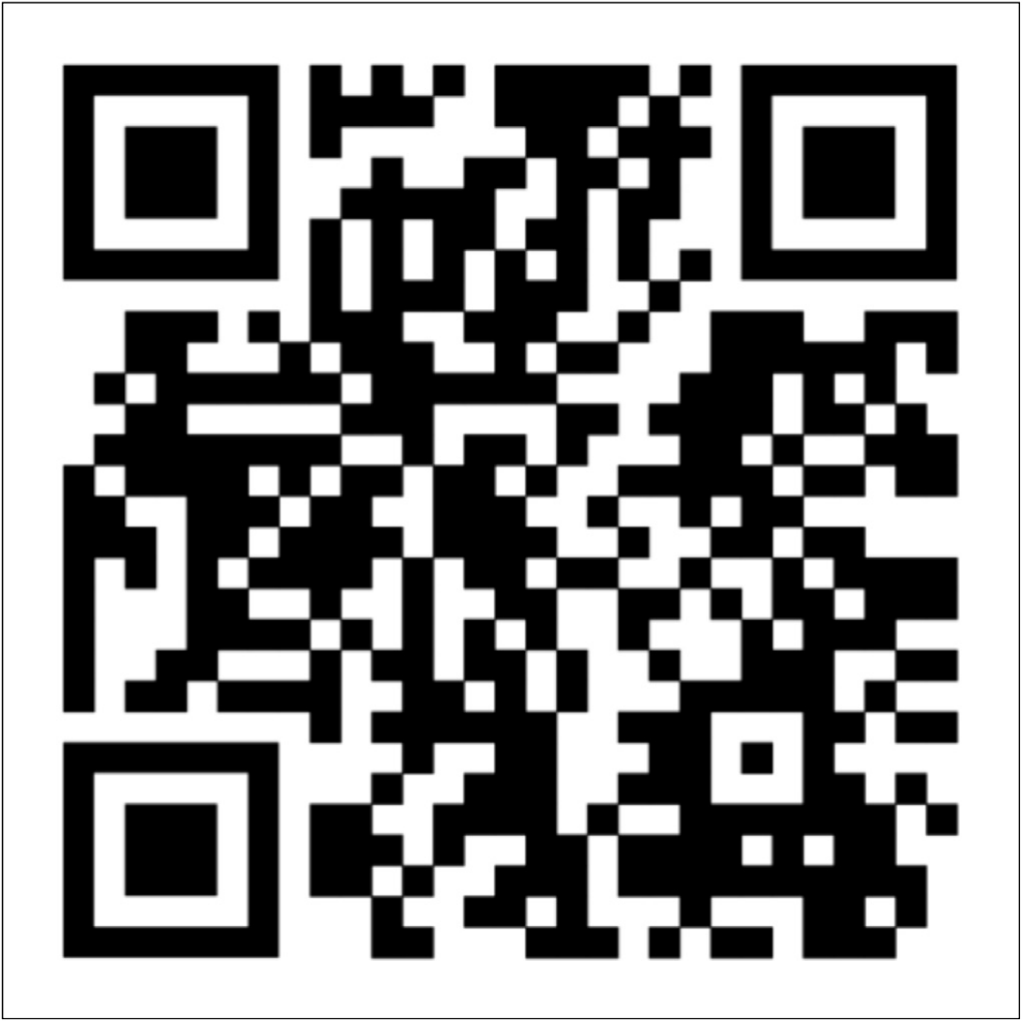
Kids-Save-Lives video. Website: www.kids-save-lives.ch. You-Tube link: https://youtu.be/fxzMOGVB8cc.

Children wanted more realistic course elements, closely relating to their world of perception. One way of addressing this request would be to let children be protagonists in the demo video to show that children are just as capable of performing CPR as adults. We therefore produced a new demo video in a public space with children as protagonists, rushes on scene, and the involvement of local police and emergency medical services (see Fig. 1). Another way of moving closer to the childrens’ world of perception could be to focus on the final scenarios. These could cover more realistic problems which children could then manage (e.g. how an unconscious person could be moved even by children), and could include more serious debriefings.

Children also wanted more excitement, especially in the video and in the final scenarios. This wish corresponds to the sensation seeking mindset of this age group.^20^, ^21^ The lighting-guided feedback from the manikins and the competitive and playful elements of the practical training obviously provided this exciting element.

### Integrating theory and practice

Schoolteachers highlighted the importance of linking theory and practice. Interestingly, children also wanted more medical information and found that integrating theory and practice was crucial. School lessons preceding the training could cover basic knowledge on cardiac arrest, thus creating a ‘blended learning’ opportunity for achieving more depth during the practical course. However, one should be aware of the tipping point where information overload begins to dilute and confuse the central learning objectives.^34^ Such effects have been observed in BLS training for adults where the teaching of the recovery position was overly emphasized in relation to more vital content, e.g., chest compressions.^35^ In this respect, schoolteachers pointed towards the necessity of stressing the overarching message of ‘*The only thing you can do wrong is doing nothing’*’, which should be constantly reinforced given the initial hesitations our interviewees expressed towards the topic. Furthermore, CPR courses for adults are often criticized for not adequately reducing the ‘fear of doing harm’,^36^ thus indicating that Kids-Save-Lives courses should put effort into enhancing the children’s self-confidence.^37^

The final scenarios were identified as an attractive course element bringing the course content to real life and at the same time encouraging the children to cooperate. Taking up the childrens’ suggestions to enhance excitement in these scenarios, it could be a helpful approach to include the schoolteachers’ expertise in this field.

### Language used

A common point was to use more consistent wording with less professional jargon. This would allow all children to better understand the course content, and a stronger focus could be put on skills teaching. In addition, it would facilitate the ability of children with migration backgrounds to follow the course content.^26^, ^38^ The teachers’ suggestion of providing a medical glossary in advance would also help address these issues.

### School teachers’ confidence

Regarding schoolteachers’ self-confidence in their ability to provide courses, the literature clearly supports their capability^39^, ^40^ and overall willingness.^41^, ^42^ However, it has also been reported that a significant barrier towards ‘Kids-Save-Lives’ training is teachers’ reluctance to deliver the training.^26^, ^42^ The teachers in our study firmely expressed the opposite opinion − after having observed the training.Thus, the ILCOR recommendation calling for schoolteachers delivering the training ^43^ may be also justified by our data.

## Limitations

This study holds inherent limitations including the single center setting, and due to the observational design, the lack of control group. However, by including all class levels, we believe that we reached sufficient information power to provide a representative picture. In addition, the design of the course was developed by experts in BLS training for adults. This may limit creativity and innovation as teachers and students were not involved in the course design from the beginning. As a further limitation, even though the children were interviewed before the teachers, an answering bias through the teachers presence still is likely. Finally, most training sessions were conducted in smaller groups which may not be possible in school classes. To account for this limitation, schoolteachers could either support each other between classes, or involve peer-students into the training.

## Implications

Overall, various points for improving schoolchildren training can be taken from our findings. To better meet the childrens’ learning needs (with their less self-regulated approach to learning),^19^ the courses should enhance realism, excitement, and add competitive elements. In addition, courses would benefit from a more curricular perspective integrating theory and practice. Theoretical content included into preceding school sessions would prepare for the skills training during the course. Finally, the teachers’ statements support the concept that teachers could deliver the courses themselves. Findings from this study could fuel the content of an urgently needed e.g., ‘ERC Schoolteacher Curriculum’ for Kids-Save-Lives training.^44^

## Conclusion

In this study, school children and their accompanying schoolteachers suggested enhancing excitement and realism and better integrating theory and practice. Incorporating these findings may help to overcome barriers to a broader dissemination of the ‘Kids-Save-Lives’ programmes.

## Conflicts of interest

**MK** is faculty of the simulation centre of the University Hospital Zurich, Switzerland, providing simulation-based training to clinicians and non-clinicians.

**DRS** (past 5 years): Dr. Spahn's former academic department is receiving grant support from the Swiss National Science Foundation, Berne, Switzerland and CSL Vifor (International) AG, St. Gallen, Switzerland.

Dr. Spahn is co-chair of the ABC-Trauma Faculty, sponsored by unrestricted educational grants from Alexion Pharma Germany GmbH, Munich, Germany, CSL Behring GmbH, Marburg, Germany, and LFB Biomédicaments, Courtaboeuf Cedex, France.

Dr. Spahn received honoraria / travel support for consulting or lecturing from:

Alliance Rouge, Bern, Switzerland, Danube University of Krems, Austria, European Society of Anesthesiology and Intensive Care, Brussels, BE, International Foundation for Patient Blood Management, Basel, Switzerland, Korean Society of Anesthesiologists, Seoul, Korea, Network for the Advancement of Patient Blood Management, Haemostasis and Thrombosis, Paris, France, Society for the Advancement of Blood Management, Mount Royal NJ, Alexion Pharmaceuticals Inc., Boston, MA, AstraZeneca AG, Baar, Switzerland, Baxter AG, Glattpark, Switzerland, Bayer AG, Zürich, Switzerland, B. Braun Melsungen AG, Melsungen, Germany, CSL Behring GmbH, Hattersheim am Main, Germany and Berne, Switzerland, CSL Vifor (Switzerland) Villars-sur-Glâne, Switzerland, CSL Vifor (International), St. Gallen, Switzerland, Celgene International II Sàrl, Couvet, Switzerland, Daiichi Sankyo AG, Thalwil, Switzerland, Haemonetics, Braintree, MA, USA, iSEP, Nantes, France, Novo Nordisk Health Care AG, Zurich, Switzerland, Octapharma AG, Lachen, Switzerland, Pharmacosmos A/S, Holbaek, Denmark, Pierre Fabre Pharma, Alschwil, Switzerland, Portola Schweiz GmbH, Aarau, Switzerland, Roche Diagnostics International Ltd, Reinach, Switzerland, Shire Switzerland GmbH, Zug, Switzerland, Werfen, Bedford, MA, Zuellig Pharma Holdings, Singapore, Singapore. (Zurich, Switzerland, January 1, 2024, Donat R. Spahn)

**JB** is Member of the ILCOR Task Force ‘Education, Implementation and Teams’, and Member of the ERC Science and Edcuation Committee ‘SEC-IES’.

**CA** and **VC-S** have no conflicts to declare.

## CRediT authorship contribution statement

**C. Andreotti:** Writing – original draft, Software, Project administration, Methodology, Investigation, Formal analysis, Data curation, Conceptualization. **M. Kolbe:** Writing – review & editing, Methodology, Investigation, Formal analysis, Conceptualization. **V. Capon-Sieber:** Writing – review & editing, Methodology, Conceptualization. **D.R. Spahn:** Writing – review & editing, Resources. **J. Breckwoldt:** Writing – review & editing, Writing – original draft, Validation, Supervision, Project administration, Formal analysis, Conceptualization.

## Declaration of competing interest

The authors declare that they have no known competing financial interests or personal relationships that could have appeared to influence the work reported in this paper.
