# Supplementary material for: Kids Save Lives – The kids’ and teachers’ view: How school children and schoolteachers would alter a BLS course designed by specialists
Source: Resusc Plus. 2024 Aug 1;19:100731. doi: 10.1016/j.resplu.2024.100731 (PMC11345691; doi:10.1016/j.resplu.2024.100731)
Supplement: Supplementary Data 1 [file mmc1.docx]

| **List of specific terms mentioned by the participants to include into a glossary** | |
| --- | --- |
| teachers: | children: |
| AED | AED |
| chest compressions | chest compressions |
| electric shock, ‘to flash’ | defibrillator |
| defibrillator |  |
| resuscitation |  |

**Supplemental material** 1:
